# Quality indicators for rural surgical and obstetrical care: A modified Delphi consensus study

**DOI:** 10.1371/journal.pone.0334143

**Published:** 2025-10-13

**Authors:** Anshu Parajulee, Abdo Souraya, Nancy Humber, Sean Ebert, Kim Williams, Tom Skinner, Jude Kornelsen

**Affiliations:** 1 Centre for Rural Health Research, Department of Family Practice, University of British Columbia, Vancouver, British Columbia, Canada; 2 Rural Coordination Centre of British Columbia, Vancouver, British Columbia, Canada; Bangabandhu Sheikh Mujib Medical University (BSMMU), BANGLADESH

## Abstract

**Objective:**

To identify contextually relevant indicators to measure the quality of surgical and obstetrical care in low-volume rural hospitals using a consensus-based methodology.

**Methods:**

A modified Delphi process was implemented in which participants were asked to rate the priority of proposed evaluation metrics over two rounds. Two Delphi surveys were electronically administered in 2019, approximately one month apart. Fifty-one health care professionals from across Canada, including rural proceduralists and quality improvement experts, were invited to participate. All quality measures in the first round were proposed by the study team. The second round included measures that did not reach consensus in the first round and measures suggested by respondents during the first round.

**Results:**

Thirty individuals participated in Round 1 (59% response rate). Of the 30 respondents from Round 1, 23 participated in Round 2 (77% response rate). 115 of 177 proposed measures (65%) reached positive consensus in Round 1 or 2. Expert participants agreed that these measures should be prioritized/included when evaluating surgical and/or obstetrical quality in rural hospitals. No measure reached negative consensus in either round. Open-text comments offered practical guidance on how to interpret and use surgical and obstetrical quality data within a rural context. Many respondents believed that rare adverse outcomes have low relevance at rural hospitals where volumes are low, procedures are almost all lower complexity day cases (Cesarean section being the major exception), and patients are typically healthy.

**Conclusion:**

The modified Delphi process resulted in the identification of surgical and obstetrical quality indicators that are contextually embedded in the realities of rural practice. The methodology allowed for the consideration of factors often overlooked by normative urban-based approaches, including team-based care characteristic of rural hospitals and limited access to specialist care and imaging services.

## Introduction

Challenges to evaluating health care quality in rural, low-volume settings have been widely identified and broadly include data availability, data quality, and data analysis [1,2]. Low data volumes reflective of low procedural volumes limit the ability to detect statistically significant differences and, consequently, preclude the ability to compare to appropriate reference groups [3]. This can be exacerbated by the heterogeneity of the population and the attendant difficulty of identifying appropriate cohorts of comparison [3]. Additionally, key health care professionals in low-volume settings often carry a high level of clinical or administrative responsibility, limiting local involvement in data collection and analysis [4]. Lack of rural sensitization can lead to the use of statistical methods typically used in high volume settings, but inappropriate for low volume settings, or the application of indicators of little relevance to procedural care in lower resourced environments [3].

The potential dissonance between urban-derived processes and the realities of rural procedural care necessitates the development of quality of care indicators that are embedded in the realities of rural practice. Rural generalist proceduralists often favour team-based metrics [5] focused on process-based assessments of quality, rather than metrics based exclusively on outcomes [3]. Process measures are more valuable for the overall assessment of quality than low-frequency, high-acuity harm indicators [3].

Historically, rural services have had limited access to local outcomes data and, consequently, limited strategies to track and review quality benchmarks, outflow, unmet needs, and patient experience. The key to any quality system is the ability of local teams to apply the philosophies of a learning system to local programs to continue to improve and adapt to population health needs and engage in local quality improvement. The study jurisdiction lacks integrated, robust systems aside from intermittent quality assurance programs, privileging dictionaries [6], and patient complaints.

The current study was completed as part of the provincially funded Rural Surgical and Obstetrical Networks (RSON) initiative in British Columbia, Canada. RSON was an integrated suite of interventions designed to address the instability of low-volume surgical and obstetrical services across rural communities. It included funding for increased scope and volume, clinical coaching, continuous quality improvement, and remote presence technology, underscored by a robust and comprehensive evaluation of processes, health outcomes, and costs [7]. The immediate challenge for the health outcomes evaluation, beyond low procedural volume across a disparate skillset, was determining relevant and helpful indicators to measure the quality of care.

Some hospitals in British Columbia [8] and elsewhere with high volumes of surgeries participate in the National Surgical Quality Improvement Program (NSQIP), a measurement system for surgical quality managed by the American College of Surgeons [9]. This system was considered for the RSON evaluation but deemed inappropriate for low-volume rural hospitals with lower acuity procedures, limited resources, and healthy patients. Further, NSQIP indicators did not include some of the most common procedures performed in rural operating rooms (ORs) (e.g., Cesarean section). Given that few studies have investigated rural surgical or obstetrical quality, and those that have, focused on specific procedures (e.g., appendectomy [10]), we identified a need for a consensus-based approach that harnesses the collective input of experts for this understudied area. To this end, we undertook a modified Delphi process with provincial and national key experts to collaboratively determine indicators that would most accurately and appropriately reflect the quality of surgical and obstetrical care within the rural hospitals participating in the RSON initiative. This process was foundational to the RSON evaluation, including a comprehensive analysis of patient health outcomes.

The Delphi method is a structured, iterative process that utilizes expert opinion to drive consensus on topics with no or minimal clear evidence on efficacy [11]. It typically involves rounds of surveys, the results of which are analyzed and reported back to panel members who can revise their ratings based on group prioritization [11]. The process continues until consensus is reached on the proposed indicators [11]. It is recognized as a reliable collaborative decision-making methodology in the absence of international best practice literature [12].

Interest in Delphi techniques to inform the surgical quality literature is burgeoning [13–19]. Despite the methodological concerns raised by some authors, namely, the unclear recommendations around sample size [13,14,18], quasi-anonymity [11], risk of ambiguous interpretations by participants [18], and the lack of standardized techniques, particularly in defining consensus [20,21], Delphi processes are increasingly recognized as a robust methodological approach. Keeney et al. [11] suggest that in addition to eliminating group bias, the repeated nature of the survey helps to establish high reliability as well as face and content validity. Delphis are also not limited by the geographical and scheduling disharmonies of its expert panel [14,18,21]. The Delphi process has several advantages when applied specifically to the development of rural procedural indicators. This includes the inherent filters to ensure the relevance and practicality of indicators for rural settings, underscored by experts’ contextual awareness of key factors such as resource availability, procedural volume, data availability, and data quality [22].

To our knowledge, no study from a high-income country has used the Delphi technique to establish quality of care indicators for rural surgical and obstetrical services. Relevant to rural obstetrical care, Rich et al. [23] used a two-round survey-based Delphi process to identify quality of care indicators for maternal care in the circumpolar region (Northernmost communities of Canada, Finland, Russia, etc.), acknowledging the need for perinatal performance indicators that are contextually relevant. A panel of 14 experts, representing diverse professions and locales, rated proposed indicators across four criteria: importance, circumpolar relevance, validity, and reliability.  Five rural/remote specific indicators were highly rated, i.e., received high ratings from > 80% of panellists. Travel to place of birth and skilled birth attendant in a patient's community were part of a group of 11 indicators that were highly rated for all four criteria and considered "core" performance indicators. Transfer for an obstetrical indication, maternity care provider in a patient's community, and uplanned birth in the community were part of a group of 29 indicators that were highly rated for the importance and relevance criteria but not the validity and/or reliability criteria, and for which the study team recommended further research and indicator development.

Guided by Donabedian’s framework for surgical care quality [24], the current study aimed to apply a modified Delphi consensus method to develop a list of rurally important surgical and obstetrical quality indicators within the context of an evaluation of British Columbia’s RSON initiative. Donabedian’s framework for quality consists of three dimensions of care: structures, processes, and outcomes. Structures relate to the setting in which care is provided, processes refer to the care patients receive, and outcomes are the events following care. Although indicators were developed with an awareness of the local contextual realities in Canada, we assert that the Delphi-derived indicators may be useful for other jurisdictions.

## Methods

We operationalized the Delphi technique in alignment with previous applications of the Delphi to identify surgical and obstetrical quality of care indicators.

### Proposed measures

In several studies, initial lists of indicators were derived from literature searches [15,25–27] or based on existing obstetrical quality measures developed by different maternity units [28]. Key considerations for the selection of indicators in the studies reviewed included validity [23,26] and feasibility [25–28]. Other desired characteristics included relevance to patient outcomes, [27] and the usefulness of measures for quality improvement [25].

In our study, measures included in the first Delphi survey were identified by study team members through a literature review of outcome and structure indicators [25,29–39].^,^ In addition to general surgical and obstetrical outcomes, we included perinatal outcomes and outcomes specific to four index surgeries/procedures commonly performed across RSON hospital ORs: Cesarean section, appendectomy, hernia repair, and colonoscopy. We did not include any process measures in the survey because they are not routinely collected at RSON hospitals, and it was deemed not feasible to collect this type of data for the RSON initiative.

Before the first Delphi round, rural physicians from the RSON initiative reviewed the initial list of indicators developed by the study team and provided feedback based on their clinical experience in rural British Columbia. Indicators were revised, removed, or added during this review stage.

### Participants

In other similar Delphi studies, panelist recruitment occurred through purposive sampling [23,40] or through recommendations by relevant expert groups [25]. In obstetrical studies, various professions were represented, including obstetricians, midwives, anesthetists [25,41], pediatricians [26,40], policymakers, and members of the public [28,40]. In surgical studies, surgeons, nurses [15,42], and anesthetists [42] contributed to the panels.

We used purposive sampling to identify rural health care providers and quality improvement experts to recruit. Each identified individual was in the personal professional network of one of the study team members, who emailed the individual to invite them to the Delphi process. The study team is comprised of rural health researchers and RSON initiative leads, some of whom are rural physicians in BC and experts in quality improvement.

Only individuals meeting specific criteria were invited to participate. They needed to have extensive clinical and/or quality improvement experience in surgical, obstetrical, and/or maternal care. As the study aimed to inform the evaluation of the RSON initiative, invitees needed to be based in Canada. They also needed to be knowledgeable about rural health services. Invitees not based in a rural community needed to be supportive of family physicians with enhanced surgical, obstetrical, and/or anesthetic skills who perform low-acuity procedures in small-volume ORs in rural Canada. The study team created a heterogeneous list of 51 invitees with diverse geographic and professional backgrounds. This heterogeneity helped to reduce bias and increase generalizability across geographic contexts. See the Results section for more information on Delphi invitees.

### Survey administration and analysis

A concurrent mixed methods study design was used [43,44]. Both quantitative and qualitative data were collected through a survey-based two-round modified Delphi process. Participants rated suggested measures and were able to supplement their ratings with comments. The two types of data were collected in the same surveys, analyzed separately, and integrated during final interpretation [44]. This design allowed for a more comprehensive understanding of experts’ perspectives on the importance of suggested measures for the rural context.

A few similar studies have used a modified Delphi technique that included a face-to-face ‘consensus meeting’ to discuss the rationale for including or excluding certain indicators [27,28] and resolve any concerns, such as language and indicator definition [26]. A synchronous meeting with participants was not feasible for our study. The two Delphi rounds in our study occurred in 2019, with three weeks between the end of the first round and the start of the second round. An email invitation was sent on July 9, 2019, for the Round 1 survey and on August 16, 2019, for the Round 2 survey. In each round, respondents had two and a half weeks to complete the survey and received up to two email reminders. Data collection ended on July 26, 2019, for the Round 1 survey and on September 4, 2019, for the Round 2 survey.

**Fig 1:**
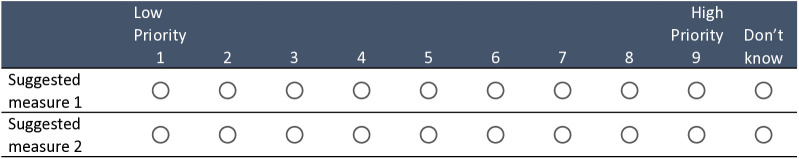
Delphi survey response scale.

The first Delphi survey included 138 outcomes and 24 structures relevant to surgical and/or perinatal care. Respondents rated measures identified by the study team and were able to suggest any outcome or structure measure not included in the survey. In each Delphi round survey, participants received the following instruction: “For each measure listed, please select its priority as an indicator (using a Likert scale from Low (1) to High (9)) for assessing the quality of surgical care for procedures performed by family physicians with enhanced surgical skills across rural facilities in British Columbia”. Participants were able to select a ‘don’t know’ response if they were not able to evaluate a suggested measure. See Fig 1 for the response scale and S1 and S2 Tables for all the suggested measures included in the Delphi process. Each measure appeared in Round 1 and/or 2. Participants were able to provide comments to supplement their ratings. Other researchers have examined more specific aspects of surgical/obstetrical indicators such as relevance, interpretability, or actionability. To minimize survey burden for busy professionals, we asked Delphi participants to only rate the overall priority of each measure when evaluating rural care quality.

The survey for Round 2 included any measure that did not reach positive or negative consensus in Round 1, and any measure suggested during Round 1 that the study team deemed relevant and for which data collection would be feasible at RSON participating hospitals. In Round 2, respondents were again asked to rate priority using a 1–9 Likert scale. For each measure, respondents were provided with their own rating from Round 1 along with two aggregate-level summary statistics from Round 1 (median and interquartile range). In Delphi processes, participants are typically provided with their own as well as other participants’ responses from a previous round to promote group convergence [11,45]. Participants can use this information when deciding if they would like to change their ratings. If a respondent provided a ‘don’t know’ response in Round 1, they were asked to provide the same response in Round 2. The criteria below were used to determine positive or negative consensus for each suggested measure. These criteria have been used by other researchers during Delphi processes [23,46].

- Positive consensus: ≥ 80% gave a measure a high rating, defined as a 7, 8, or 9 rating.
- Negative consensus: ≥ 80% gave a measure a low rating, defined as a 1, 2, or 3 rating.

Quantitative data were analyzed using Microsoft Excel, and qualitative data were analyzed using NVivo. ‘Don’t know’ responses were excluded from quantitative analyses. Respondent comments were analyzed inductively, and thematic areas are described narratively [47].

### Ethics

This study obtained ethical approval from the University of British Columbia’s Behavioural Research Ethics Board (UBC CREB) (H19-00950). A consent form, which appeared at the beginning of each Delphi survey, stated that electronic submission of the survey signified that respondents had provided their consent to participate in the Delphi process. Participants were also informed that once the study’s findings are made publicly available (e.g., conferences, academic publications), they would not be able to withdraw their participation. The contact information of three study leads and UBC CREB (emails and telephone numbers) was provided if participants had any questions or concerns, or wanted to withdraw from the study. As the identities of respondents were needed to report each respondent’s ratings back to them in the Round 2 survey, data were collected confidentially rather than anonymously. The collection of respondent identifiers also allowed for an assessment of respondent heterogeneity. Data were collected electronically through the online UBC Qualtrics platform.

## Results

Thirty of the 51 individuals invited to complete the Round 1 survey did so (59% response rate). Twenty-three of the 30 individuals who completed the Round 1 survey and were invited to complete the Round 2 survey did so (77% response rate). The percentage of respondents based in a rural community was 80% in Round 1 and 78% in Round 2. See Table 1 for more information on the characteristics of invitees and respondents.

**Table 1:** Characteristics of Delphi invitees and respondents.

	Invitees n (%)	Round 1 respondents n (%)	Round 2 respondents n (%)
**Region**			
British Columbia	43 (84)	24 (80)	18 (78)
Other province/territory in Canada	8 (16)	6 (20)	5 (22)
Total	51 (100)	30 (100)	23 (100)
**Background**			
RSON hospital – Rural physician*	18 (35)	13 (43)	8 (35)
RSON hospital – OR nurse	3 (6)	2 (7)	2 (9)
Referral RSON hospital – Specialist physician^ǂ€^	9 (18)	3 (10)	3 (13)
Other rural hospital – Rural physician*	7 (14)	6 (20)	5 (22)
Non-rural hospital – Specialist physician^ǂ^	7 (14)	3 (10)	3 (13)
Physician quality improvement lead (in British Columbia)^¥^	7 (14)	3 (10)	2 (9)
Total	51 (100)	30 (100)	23 (100)

* Physician who provides maternity care and/or care in the OR. ^ǂ^ Physician specialized in surgery, gastroenterology, obstetrics/gynecology, or anesthesiology. ^€^ Referral RSON hospitals can also be rural hospitals. These hospitals serve larger local populations, have a wider scope of services, and are considered a higher level of care relative to RSON hospitals. ^¥^ Two of these leads were based in rural communities but neither participated in the study.

### Quantitative

**Fig 2:**
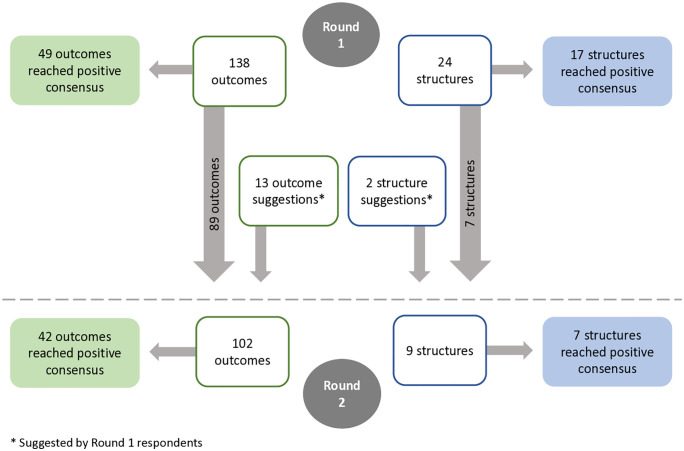
Modified Delphi process and results.

Overall, across the two Delphi rounds, 115 of the 177 measures suggested by study team members or Delphi respondents reached positive consensus (65%). The percentage of measures reaching positive consensus was approximately the same across rounds: 41% in Round 1 and 44% in Round 2. A majority of proposed outcome measures (91/151; 60%) and most structure measures (24/26; 92%) reached positive consensus. Only two of the 15 measures suggested by respondents, both outcomes, reached positive consensus in Round 2. No measure reached negative consensus in either round. Fig 2 provides an overview of the Delphi process and results.

The outcomes and structures that reached positive consensus are listed in Tables 2–4. At least 80% of expert participants agreed that these measures should be prioritized/included when evaluating surgical and/or obstetrical care quality in rural hospitals. Of note, six measures received a high rating from all respondents (in Round 2): transfer to a higher level of care, major perineal tear, transfer to NICU I, II or III, and wound dehiscence after Cesarean section. Refer to S1 and S2 Tables for the percentage of respondents who gave a ‘high’ rating for each proposed measure in each round. Several measures were close to reaching positive consensus (≥75% high ratings) but did not meet the pre-determined threshold of 80%.

**Table 2:** Adverse outcomes that reached positive consensus.

General	
**General** Death Retained foreign body part Transfer to a higher level of care Transfer to intensive care unit Unplanned readmission to hospital within 30 days Unscheduled return to OR Wound disruption **Anesthesia** Aspiration pneumonia Documented adverse drug reaction Failed regional anesthesia Failed tracheal intubation Intraoperative cardiac dysrhythmia/arrest Medication error – Wrong dose Medication error – Wrong medication Problem with airways in post-anesthesia recovery Respiratory depression Severe hypotension Transfer to intensive care unit/higher level of care during post-anesthesia recovery	**Cardiac** Cardiac pulmonary resuscitation Myocardial infarction Myocardial infarction, ST-elevation **Hematological** Blood transfusion Deep vein thrombosis Pulmonary embolism **Infection related** Sepsis Septic shock Surgical site infection Superficial Deep Organ **Respiratory** Ventilator used within 48 hours Unplanned intubation
**Perinatal and procedure specific**	
**Appendectomy** Abscess drainage Bladder injury Bowel injury Gastrointestinal complication Laparotomy (repeat) Reoperation Systemic complication **Colonoscopy** Bowel perforation Heavy bleeding post-procedure Missed colorectal cancer **Hernia repair** Bladder injury Bowel injury Failure to be daycare surgery Hernia reoccurrence Reoperation **Cesarean section** Bladder injury Bowel injury Endometritis Intraoperative hysterectomy Postpartum wound infection Retained placenta Wound dehiscence	**Maternal** Abscess/hematoma (among those with an epidural) Major perineal tear Postpartum hemorrhage Uterine rupture **Neonatal** Birth trauma Death Infant (1–12 months) Late neonatal (<1 month) Perinatal Resuscitation at birth Total length of stay in neonatal intensive care unit (NICU) (days, among infants in NICU) Transfer to NICU NICU I NICU II NICU III Composite – Adverse outcome index

**Table 3:** Non-adverse outcomes that reached positive consensus.

**General** Patient reported outcomes Patient reported experience **Appendectomy** (pathology or intervention related) Appendicitis Associated imaging completed Laparoscopic Pathology confirmed appendicitis Perforated appendicitis **Cesarean section** (type) # of Cesarean sections: Total Planned Planned, repeat, second for mother Planned, repeat, ≥ 3 for mother Emergency	**Colonoscopy** Adenoma detection Adenoma resection rate for polyps <20 mm in size* Adenoma retrieval Cancer detection Cecal intubation Fecal immunochemical test (FIT) Colonoscopy completion rate after FIT positive result FIT wait time to follow-up colonoscopy (days) **Maternal** Antepartum length of stay (days) Assisted vaginal delivery (forceps or vacuum) Logistics as reason for induction Postpartum length of stay following vaginal delivery (days)

* Several respondents commented that 20 mm is large for a rural context; large polyps are typically referred to a higher level of care for removal. Suggestion to change to <10 mm in size.

**Table 4:** Structures that reached positive consensus.

**OR personnel** Number of Family physicians with enhanced surgical skills (FP ESS) Family physician anesthetists (FPAs) Family physicians with obstetrical surgical skills (FP OSS) General surgeons OR nurses OR nurses available on call **24/7 OR call for** Nursing Anesthesiology Surgery **Maternity personnel** Number of Maternity nurses Midwives Primary care doctors providing maternity care	**Volume and OR days** Mean monthly surgical volume Mean monthly obstetrical volume Proportion of daytime operations FP ESS mean monthly surgical volume Number of OR days per week **Volume and OR days, visiting specialists** Mean monthly number of procedures Mean monthly number of OR days used Types of procedures completed **Equipment** Ultrasound equipment available on site Ultrasound technician available in the community **Times** Median time to theatre (for acute cases) Wait time (from surgery booking to surgery date)

### Qualitative

Respondent comments provided further insight into the relevance and utility of suggested measures and offered practical guidance on how to use data within a rural context. Many respondents believed that rare adverse outcomes have low relevance at rural hospitals where volumes are low, procedures are almost all lower complexity day cases, and patients are typically healthy. There was the assertion by several that if a rare event were to occur, there would be immediate investigation and follow-up, both locally and regionally, through processes such as Patient Safety Learning Systems case review and patient complaint systems.

*“Typically, in this rural setting, most of our ASA’s [American Society of Anesthesiologists’ Physical Status Classification System] are 1 and 2 with the occasional 3 depending on the procedure. We don’t see many of the outcomes listed in this survey, but we follow up if there are any.” – Respondent 14*

Related to relevance, several respondents thought collecting data on rare events at low-volume sites would have low utility given that it would be difficult to achieve sufficient statistical power for comparisons across time or sites for quality improvement or research purposes. Nonetheless, a few believed it important to collect data on certain commonly reported indicators, even if they are very rare, for instance, blood transfusion for maternity care and deep vein thrombosis. One respondent explained that an exception to the low volume challenge in rural British Columbia is colonoscopy as hospitals have high enough volumes to allow for comparison with “any endoscopy unit anywhere”.

Some were concerned about the potential negative consequences of collecting data on very rare outcomes. For example, if a severe adverse outcome were to occur, the prevalence of this outcome would appear high for many years due to low annual volumes, potentially leading to a perception that rural surgical programs provide poor quality care. Another concern was inefficient use of limited resources. Several respondents recommended focusing on more common outcomes such as surgical site infection and pneumonia.

*“It may take years at our site for this kind of event to happen. How do you use any of these measures which may have validity when looked at over 100,000 or a million procedures at a site that does less than 1000 procedures in a year? … One bad outcome spread over all of our sites would provide horrible looking data for years.” – Respondent 17*

A few thought that some Delphi measures reflect the broader health care system rather than surgical quality at specific sites. For instance:

*“Suggest separating those outcomes due to the procedure and the skill with which it [procedure] is performed and those due to system factors - i.e., those related to FIT [fecal immunochemical test].” – Respondent 4*

Although one respondent believed it is often difficult to predict whether an adverse event will occur and that “it is more important how they are dealt with rather than their occurrence”, several others thought their occurrence may point to a need to review case selection processes, particularly from an anesthetic perspective. Relatedly, one respondent described rating adverse events in the Delphi survey based on preventability.

*“These [outcomes] are all important and may reflect surgical and anaesthetic decision-making (i.e., appropriateness of patient for a rural site vs how the event was managed if it did occur in an appropriately selected patient).”– Respondent 15*

A few respondents thought it would be useful to compare local site data to aggregate provincial data. In such a scenario, there was a suggestion to investigate any outlier data points and follow up locally if necessary. For instance:

*“I think these are all valuable measures to assess; if a program is outside of range compared to similar communities (either too often or not enough), it could be a useful tool to assess the quality at that site (i.e., site with few vacuum deliveries, should they go to a higher volume site for CPD [continuing professional development] on that skill? If one site is never inducing, are they sending them out and could be supported in keeping more care at home, etc.).” – Respondent 15*

Some described contextual factors that need to be considered when reviewing data and comparing rural sites to referral sites. There was the expectation that referral sites with higher acuity cases would have higher complication rates. One respondent believed that rural sites will typically have longer operative length times, but that this difference does not necessarily need to be viewed as problematic. Another believed it would be "unfair" to expect rural sites, where access to specialized technology is limited, to have the same diagnostic accuracy and imaging rates as referral sites. Regarding structure measures, several emphasized various challenges that rural sites can face, such as the lack of local access to ultrasound, difficulty minimizing disruptions to 24/7 on-call schedules when teams are small, and only being able to perform elective appendectomies due to the lack of local imaging to confirm appendicitis.

*“The main OR program killer will always be burden of ON CALL hours required for doctors and OR nurses.” – Respondent 17*

A few respondents were unsure how to interpret data for some measures, such as what epidural rate is considered appropriate and how to use composite measures for quality improvement. There was a recommendation to conduct individual case reviews to supplement composite measures. In response to suggested personnel-related measures, some pointed out that each community considers its specific needs when determining the most appropriate mix of practitioners. For instance, the view that a community does not necessarily need to have midwives, but that it would be interesting to examine across multiple rural communities the relationship between the number of midwives and the number of family physicians providing maternity care.

Eight OR volume measures were included in the Delphi survey, but one respondent did not think these measures should be used:

*“I do not believe that volume is a good proxy for quality of surgical care. At low volume and with excellent continuous quality improvement, the surgical care can still be at a high standard.” – Respondent 22*

## Discussion

The Delphi approach allowed us to take into consideration domains of rural health care often overlooked when normative, urban approaches for high-volume settings are used. Most important is the health human resource complement, which, in our study jurisdiction, involved procedural care by local family physicians with enhanced surgical or obstetrical skills, supported by family physician anesthetists, as well as outreach specialists from larger centers. The objectives of generalist procedural education and training programs are to equip rural providers with a skill set that allows safe practice for low-acuity procedures in low-acuity patients [48], thereby enhancing access to local care. Historically, however, there has not been widespread interest in rigorous evaluation of the quality of these models of care, particularly across a wide domain. The stepwise process we used to develop consensus-based indicators contributed both to the face validity of our final analysis for the RSON evaluation [49] and to establishing a foundation for assessing rural procedural quality.

Aside from general procedural quality markers such as death, wound disruption, or myocardial infarction, participants in this process identified contextually specific priorities for rural surgical quality measurement, such as ‘transfer to a higher level of care’, which received a high priority rating from all respondents in Round 2, reflecting the great importance of surgical and maternity triage to high-quality rural care. This variable reflects the essential rural-specific skill of determining appropriate case selection for local care and ensures that patients deemed likely to experience challenges due to comorbidities are triaged to a higher level of care for their procedure. This also reflects the essential triage function of rural teams to ensure patients receive the right care in the right place at the right time.

Another rurally specific non-adverse outcome that reached consensus in the context of obstetrical care was ‘logistics as a reason for induction’. This is a key process measure that reflects the volume of remote patients who may have traveled from outlying communities and are anxious to return home. Culturally sensitive indicators, such as minimizing time away from one’s community for Indigenous patients, are also essential rurally specific indicators of quality. Similarly, in Rich et al.'s [23] Delphi process for maternity care indicators for rural/remote circumpolar regions, experts provided high ratings for indicators typically used in urban settings (e.g., NICU admission) as well as indicators specific to rural/remote settings (e.g., travel to place of birth).

Structure indicators that reached positive consensus included key markers of service stability, including mean monthly surgical volume and number of OR days per week, as well as service enablers such as availability of on-site ultrasound machines and ultrasound technicians [50]. A measure that almost all participants were keen to see captured was surgical wait times, as this allows for the expression of the value of small sites within the larger healthcare system, where surgical wait times are significantly lower. Although not a quality indicator specific to a single site, this is aligned with the broad domains of quality, reflective of a comprehensive approach to care delivery in low-volume settings. Key to the development of a robust, rurally aligned framework is the understanding that quality indicators traditionally focus on past harm and quality assurance. Rural teams, however, are motivated to prospectively keep patients and families safe in their services.

As noted in the qualitative findings, many respondents perceived the lack of value of including rare and very rare events in the suite of indicators, given how infrequently some of them occur and the challenge of interpretability should one occur, highlighting the low patient acuity anticipated in rural sites and historical overreliance on low-value past harm and low-frequency events in rural settings. Among the rare/very rare suggested outcomes, some, such as acute coronary syndrome and blood transfusion reaction, did not reach positive consensus, but others, such as septic shock and acute organ surgical site infection did. These low-frequency, high-morbidity events are important from a process perspective and should be tracked and reviewed continuously. They enable comparisons to population health outcomes and should take into account system attributes that influence rural sites. It can also allow for a more comprehensive comparison with urban facilities, which are often considered the reference standard of care.

The need for a rural-specific framework for quality measures, driven by contextual awareness from those at the front lines, was reinforced through our study. The modified Delphi process was an effective mechanism for ensuring that quality indicators used in the RSON evaluation were identified ‘through a rural lens’. Though our study focused on sites in rural British Columbia, Canada, findings have salient implications for health care evaluation in rural jurisdictions with similar contextual factors, in the United States of America, as well as other countries. Relevant factors include sites with low procedural volume and significant distances and surface travel times to larger health centres, characteristics of rural health services internationally. As Finlayson noted in a 2009 article titled ‘Assessing and improving the quality of surgical care in rural America’:

“Current models for surgical quality assessment and improvement largely reflect the characteristics of larger urban hospital settings, which include proximity to other providers for peer review, higher procedure volumes to accurately assess outcomes, and greater financial resources to acquire data collection systems and finance participation in regional or national quality improvement programs, such as the American College of Surgeons National Surgical Quality Improvement Program.” [3 (p.1380)]

## Limitations

Although the Delphi process is effective for gaining expert-based consensus on topics with scant existing evidence, there are also known limitations, including the potential for participant bias and limited diversity of perspectives. We endeavored to select diverse panel members who were knowledgeable about rural health services and/or experienced in quality improvement, but we may have inadvertently excluded divergent viewpoints.

Other researchers may want to assess more specific aspects of proposed surgical/obstetrical quality indicators in a Delphi process, such as relevance, interpretability, or actionability. They may also choose to assess process measures and include other procedures common in their jurisdiction, such as orthopedic and dental procedures. Measures suggested by respondents during Round 1 were only rated by respondents once during Round 2. A third Delphi round may have resulted in a greater number of suggested measures reaching positive or negative consensus. Despite these potential limitations, we assert that our application of the Delphi process for determining rural surgical and obstetrical quality measures was effective and led to a more robust evaluation framework for the RSON initiative.

## Conclusion

The lack of rural-specific data is the result of challenges to data quality and access, and, most salient to the work we have undertaken, accepted metrics to appropriately assess quality in low-volume settings. The Delphi process emphasized the importance of establishing obstetrical and surgical quality indicators that are rurally relevant. Delphi participants also described the fundamental methodological challenges of including commonly reported rare adverse events when procedural volume is low, highlighting the need for rural-specific analytic approaches.

## Supporting information

S1 Table Suggested outcome measures – Percentage of positive ratings (7, 8, or 9 rating). (DOCX)

S2 Table Suggested structure measures – Percentage of positive ratings (7, 8, or 9 rating). (DOCX)

S1 Data File Delphi survey responses with (potentially) identifying information removed. (XLSX)
