# Supplementary material for: Quality indicators for rural surgical and obstetrical care: A modified Delphi consensus study
Source: PLoS One. 2025 Oct 13;20(10):e0334143. doi: 10.1371/journal.pone.0334143 (PMC12517512; doi:10.1371/journal.pone.0334143)
Supplement: S1 Table — (DOCX) [file pone.0334143.s001.docx]

**S1 Table. Suggested outcome measures - Percentage of positive ratings (7, 8, or 9 rating)**

* Indicates non-adverse measure

Green shading indicates that positive consensus was reached (in either round for the ‘Measure’ column)

| **Measure** | **Percentage of respondents providing a 7, 8, or 9 rating** | |
| --- | --- | --- |
|  | **Round 1**  **(n=30)** | **Round 2**  **(n=24)** |
| **General** |  |  |
| Death of patient | 0.92 | - |
| Length of stay - Total (days)* | 0.38 | 0.16 |
| Length of stay - Surgery to discharge (days)* | 0.46 | 0.32 |
| Pain intensity above 4 assessed 4 hours after surgery | 0.42 | 0.48 |
| Patient reported outcomes* | 0.96 | - |
| Patient reported experience* | 0.88 | - |
| Postoperative delirium | 0.40 | 0.30 |
| Retained foreign body | 0.77 | 0.81 |
| Transfer to higher level of care | 0.77 | 1.00 |
| Transfer to ICU | 0.84 | - |
| Unplanned readmission to hospital within 30 days | 0.78 | 0.95 |
| Unscheduled return to OR | 0.89 | - |
| Wound disruption | 0.62 | 0.95 |
| Composite measure - Surgical Apgar Score | 0.38 | 0.56 |
| *Measures suggested by respondents during Round 1* |  |  |
| # of patients admitted* | - | 0.53 |
| Operative length (minutes)* | - | 0.53 |
| **Cardiac** |  |  |
| Acute coronary syndrome | 0.67 | 0.72 |
| Congestive heart failure | 0.58 | 0.72 |
| Cardiac pulmonary resuscitation | 0.75 | 0.83 |
| Myocardial infarction (heart attack) | 0.71 | 0.83 |
| Myocardial infarction, NONSTEMI | 0.67 | 0.78 |
| Myocardial infarction, STEMI | 0.67 | 0.83 |
| **Hematological** |  |  |
| Blood transfusion | 0.65 | 0.85 |
| Blood transfusion reaction | 0.36 | 0.21 |
| Deep vein thrombosis | 0.69 | 0.85 |
| Pulmonary embolism | 0.76 | 0.95 |
| **Neurological** |  |  |
| Cerebrovascular accident (stroke) | 0.70 | 0.78 |
| Transient ischemic attack | 0.52 | 0.72 |
| **Renal** |  |  |
| Acute renal failure | 0.61 | 0.78 |
| **Respiratory** |  |  |
| Pneumonia | 0.67 | 0.79 |
| Ventilators used within 48 hours | 0.73 | 0.88 |
| Unplanned intubation | 0.71 | 0.84 |
| **Infection related** |  |  |
| Sepsis | 0.81 | - |
| Septic shock | 0.81 | - |
| Superficial surgical site infection | 0.73 | 0.85 |
| Deep surgical site infection | 0.92 | - |
| Organ surgical site infection | 0.85 | - |
| Urinary tract infection | 0.54 | 0.65 |
| **Anesthesia** |  |  |
| Anaphylaxis | 0.64 | 0.79 |
| Aspiration pneumonia | 0.81 | - |
| Dental trauma | 0.65 | 0.55 |
| Documented adverse drug reaction | 0.71 | 0.80 |
| Failed regional anesthesia | 0.62 | 0.80 |
| Failed tracheal intubation | 0.88 | - |
| Hypothermia in recovery period | 0.54 | 0.70 |
| Inadequate reversal of neurological blockade during recovery | 0.60 | 0.75 |
| Intraoperative cardiac dysrhythmia/arrest | 0.84 | - |
| Medication error - Wrong dose | 0.88 | - |
| Medication error - Wrong medication | 0.88 | - |
| Neurological dysfunction three months after procedure | 0.55 | 0.78 |
| Post-dural puncture headache | 0.46 | 0.53 |
| Postoperative nausea and vomiting | 0.40 | 0.47 |
| Problem with airways in post-anesthesia recovery | 0.85 | - |
| Respiratory depression | 0.64 | 0.80 |
| Severe hypotension | 0.88 | - |
| Severe pain in recovery unit | 0.69 | 0.75 |
| Unplanned post-anesthesia care unit stay longer than 2 hours | 0.46 | 0.40 |
| *Measures suggested by respondents during Round 1* |  |  |
| Transfer to ICU/higher level of care due to post anesthesia recovery | - | 0.80 |
| Administering anesthetic time (ends when surgical preparations begin) (minutes)* | - | 0.40 |
| Overall anesthesia time (minutes)* | - | 0.45 |
| **Maternal interventions*** |  |  |
| Assisted vaginal delivery (forceps or vacuum) | 0.83 | - |
| Augmentation | 0.70 | 0.74 |
| Episiotomy | 0.43 | 0.47 |
| Epidural | 0.64 | 0.67 |
| Induction | 0.70 | 0.79 |
| Logistics as reason for induction | 0.81 | - |
| **Maternal outcomes** |  |  |
| Abscess/hematoma (among those with an epidural) | 0.76 | 0.94 |
| Major perineal tear | 0.78 | 1.00 |
| Pain intensity above 4 assessed 4 hours after birth | 0.26 | 0.37 |
| Antepartum length of stay (days)* | 0.52 | 0.84 |
| Postpartum length of stay following vaginal delivery (days)* | 0.70 | 0.89 |
| Postpartum hemorrhage | 0.74 | 0.89 |
| Unplanned out of hospital birth | 0.45 | 0.44 |
| Uterine rupture | 0.82 | - |
| **Neonatal** |  |  |
| Birth trauma | 0.82 | - |
| Birth weight <2500 grams | 0.59 | 0.79 |
| Gestational age <37 weeks | 0.68 | 0.68 |
| Infant death (1-12 months) | 0.82 | - |
| Late neonatal death (< 1 month) | 0.86 | - |
| Meconium at birth | 0.32 | 0.16 |
| Perinatal death | 0.91 | - |
| Resuscitation at birth | 0.61 | 0.89 |
| Still birth | 0.59 | 0.78 |
| Total length of stay in NICU (days, among infants in the NICU) | 0.70 | 0.84 |
| Transfer to NICU | 0.83 | - |
| Transfer to NICU I | 0.72 | 1.00 |
| Transfer to NICU II | 0.71 | 1.00 |
| Transfer to NICU III | 0.72 | 1.00 |
| Composite measure - Adverse outcome index | 0.80 | - |
| *Measures suggested by respondents during Round 1* |  |  |
| Weight loss of neonate during hospital stay >10% | - | 0.74 |
| Hypoglycemia post birth (first 24 hours of life) | - | 0.68 |
| Apgar score | - | 0.79 |
| **Cesarean section** |  |  |
| Bladder injury | 0.88 | - |
| Bowel injury | 0.88 | - |
| C-sections - # total* | 0.83 | - |
| C-sections - # Planned* | 0.88 | - |
| C-sections, planned - # repeat, second for mother* | 0.78 | 0.89 |
| C-sections, planned - # repeat, ≥3 for mother, * | 0.70 | 0.83 |
| C-sections - # emergency* | 0.96 | - |
| Endometritis | 0.65 | 0.83 |
| Intraoperative hysterectomy | 0.87 | - |
| Postoperative ileus | 0.35 | 0.17 |
| Postpartum length of stay following Caesarean delivery (days)* | 0.61 | 0.78 |
| Postpartum wound infection | 0.75 | 0.94 |
| Retained placenta | 0.65 | 0.88 |
| Wound dehiscence | 0.79 | 1.00 |
| *Measures suggested by respondents during Round 1* |  |  |
| Use of spinal anesthesia* | - | 0.72 |
| Use of general anesthesia* | - | 0.78 |
| Decision to incision time (for emergency C-sections) – Time from decision to operate to operative incision* | - | 0.76 |
| **Appendectomy** |  |  |
| Abscess drainage | 0.68 | 0.82 |
| Appendicitis* | 0.78 | 0.89 |
| Associated imaging completed* | 0.62 | 0.87 |
| Bladder injury | 0.84 | - |
| Bowel injury | 0.84 | - |
| Discharge – routine* | 0.48 | 0.42 |
| Duration of post-antibiotic treatment (days)* | 0.43 | 0.53 |
| Gastrointestinal complication | 0.57 | 0.89 |
| Generalized peritonitis | 0.70 | 0.78 |
| Laparoscopic* | 0.65 | 0.89 |
| Laparotomy (repeat) | 0.88 | - |
| Pathology confirmed appendicitis* | 0.80 | - |
| Perforated appendicitis* | 0.64 | 0.89 |
| Reoperation | 0.96 | - |
| Systemic complication | 0.88 | - |
| *Measures suggested by respondents during Round 1* |  |  |
| Decision to procedural start time – Time from admission to emergency department bed to operative incision* | - | 0.40 |
| **Hernia repair outcomes** |  |  |
| Bladder injury | 0.86 | - |
| Bowel injury | 0.90 | - |
| Failure to be daycare surgery | 0.70 | 0.81 |
| Gastroenteritis | 0.20 | 0.08 |
| Gastrointestinal bleed | 0.40 | 0.29 |
| Hematoma/seroma | 0.45 | 0.56 |
| Hernia reoccurrence | 0.83 | - |
| Neuralgia | 0.62 | 0.75 |
| Orchitis | 0.65 | 0.71 |
| Recurrence of pain >30 days post-operation | 0.60 | 0.73 |
| Reoperation | 0.95 | - |
| Scrota abscess | 0.75 | 0.75 |
| Urinary retention | 0.38 | 0.47 |
| **Colonoscopy** |  |  |
| Adenoma detection* | 0.96 | - |
| Adenoma resection rates for polyps <20 mm in size* | 0.85 | - |
| Adenoma retrieval* | 0.85 | - |
| Bowel perforation | 0.93 | - |
| Cancer detection* | 0.85 | - |
| Cecal intubation* | 0.96 | - |
| Fecal immunochemical test (FIT) - Colonoscopy completion rate after FIT positive* | 0.88 | - |
| FIT Wait time to follow-up colonoscopy (days)* | 0.79 | 0.94 |
| Heavy bleeding post-procedure | 0.85 | - |
| Postoperative pain | 0.48 | 0.55 |
| Sedation score* | 0.46 | 0.55 |
| Withdrawal time (minutes)* | 0.76 | 0.79 |
| *Measures suggested by respondents during Round 1* |  |  |
| Missed colorectal cancer | - | 0.90 |
