# Supplementary material for: Quality indicators for rural surgical and obstetrical care: A modified Delphi consensus study
Source: PLoS One. 2025 Oct 13;20(10):e0334143. doi: 10.1371/journal.pone.0334143 (PMC12517512; doi:10.1371/journal.pone.0334143)
Supplement: S2 Table — (DOCX) [file pone.0334143.s002.docx]

**S2 Table. Suggested structure measures - Percentage of positive ratings (7, 8, or 9 rating)**

Green shading indicates that positive consensus was reached (in either round for the ‘Measure’ column)

| **Measure** | **Percentage of respondents providing a 7, 8, or 9 rating** | |
| --- | --- | --- |
|  | **Round 1**  **(n=30)** | **Round 2**  **(n=24)** |
| **OR personnel** |  |  |
| # of … |  |  |
| family physicians with enhanced surgical skills (FP ESS) | 0.86 | - |
| family physician anesthetists (FPAs) | 0.86 | - |
| family physicians with obstetrical surgical skills (FP OSS) | 0.86 | - |
| general surgeons | 0.67 | 0.85 |
| OR nurses | 0.89 | - |
| OR nurses available on call | 0.93 | - |
| 24/7 OR call for … |  |  |
| nursing | 0.89 | - |
| anesthesiology | 0.89 | - |
| surgery | 0.89 | - |
| *New measures* | | |
| # of obstetricians | | 0.71 |
| # of permanent specialists | | 0.68 |
| **Maternity personnel** | |  |
| # of … |  |  |
| maternity nurses | 0.92 | - |
| midwives | 0.57 | 0.81 |
| primary care doctors providing maternity care | 0.92 | - |
| **Volume and OR days** |  |  |
| Mean monthly surgical volume | 0.85 | - |
| Mean monthly obstetrical volume | 0.85 | - |
| Proportion of daytime operations | 0.73 | 0.85 |
| FP ESS mean monthly surgical volume | 0.85 | - |
| # of OR days per week | 0.74 | 0.90 |
| **Volume and OR days, visiting specialists** |  |  |
| Mean monthly # of procedures | 0.78 | 0.85 |
| Mean monthly # of OR days used | 0.74 | 0.85 |
| Types of surgical procedures completed | 0.89 | - |
| **Equipment** |  |  |
| Ultrasound equipment available onsite | 0.96 | - |
| Ultrasound technician available in the community | 0.81 | - |
| **Times** |  |  |
| Median time to theatre (for acute cases) | 0.81 | - |
| Wait time (surgery booking 🡪 surgery date) | 0.78 | 0.95 |
